# Medical Education and Legislation

**Published:** 1875-08

**Authors:** 


Medical Education and Medical Legislation.—The period of the annual recurrence of medical college commencements is always the occasion for diatribes, in medical and secular journals, upon the defects of "the modern system of medical education," the "low standard of preliminary acquirements," the "superficial character of the instruction imparted to medical students," and various other disadvantages which the American, and especially the Western, medical student is supposed to sustain, in comparison with his more fortunate compeer on the other side of the ocean. The stereotyped facetiæ about "crops of sawbones," "licenses to kill," etc., occupy their usual places in the "funny columns" of the press. The critics are not, however, altogether merciless, for while indicating the faults, they are charitable enough to designate the remedy, and this they find in that great panacea of the untutored mind, "the law." The grand catholicon under whose magical influence all these evils are to disappear, is "Medical Legislation." Teachers are to be made competent and conscientious, students instructed and accomplished, physicians learned and skilful, by acts of legislature.

Lord Thurlow said that an "act of Parliament could not be drawn through which he could not drive a coach and horses." Might not a medical student be driven through an "Act of Legislature?" Legislation, then, appears to be, in the eyes of the reformers, the means, and the only means, by which the medical profession is to be brought "up to the level of the legal and clerical," with each of which it is now most discreditably compared.

We assert boldly, that were the great mass of general practitioners required to pass the examination to which medical students are now subjected, and which is yearly becoming more rigid, the percentage of rejections would be far greater than in an average class of candidates for graduation in many of the medical colleges. Don't condemn the graduate of to-day until you have measured your acquirements with his. All that is old is not venerable. There are antique as well as modern shams, and the "....laudator temporis acti" lived too in the days of Horace.

In the opinion of many, these evils may all be effectually remedied by the establishment of a State Board of Examiners, whose certificate should be not only *prima facie* evidence, but proof of the competency of medical men. The idea is plausible and superficially captivating, but will it justify our indorsement when critically analyzed? In the first place, we fail to perceive in what respect the certificate of a State Board of Examiners will be more positive evidence of competency than the diploma of the faculty of a college, as the latter would be, in all probability, the larger body of the two, and equal at least to the former in professional standing, being composed of physicians deemed by their colleagues—the most competent tribunal—peculiarly fitted for their positions.

Faculties of medical colleges are always, to say the least, up to the average of their profession in point of ability and professional acquirement, and usually above it, never below. Can the same be said of "Boards" consisting in whole or in part of medical men?

How could these Boards be appointed? by the Governor? that is to say, by personal influence; by the Legislature? that is to say, by political influence; or by the people? that is to say, by popular influence. Than the first of these there could hardly be a more unreliable method of choosing the fittest, except the second and third, and the same might be said of each of the other methods respectively. The Governor would appoint his own physician and his friends, or suffer an imputation upon his own judgment. The Legislature would appoint those having most political influence, in the acquisition of which no man ever became an accomplished physician. And the people would elect the most popular doctors, who would generally be found to have cultivated popularity, to the exclusion of science. * * *

We have seen several of these projects for the establishment of "State Boards," and—while we have no desire or intention to stigmatize all as such—these were all nothing more nor less than contrivances by which somebody, unable or unwilling, or both, to attain professional position by hard work, sought to acquire some temporary notoriety, a small stipend, and "the chances," at the expense of the State.

In conclusion, we assert our most thorough sympathy with those who desire the elevation of the standard of professional education, but cannot indorse all the utopian schemes which have been devised for the accomplishment, believing the only reasonable plan—which must eventually succeed—to be the silent force of example. Let every critic and reformer study diligently and faithfully to become truly expert in some department of his profession, and all who come within the range of his influence will be stimulated to follow his example—and so the standard of medical education will be raised *tuto, cito, et jucunde.—Chicago Medical Journal.*

